# Axial impairment and falls in Parkinson’s disease: 15 years of subthalamic deep brain stimulation

**DOI:** 10.1038/s41531-022-00383-y

**Published:** 2022-09-24

**Authors:** Alessandro Zampogna, Francesco Cavallieri, Francesco Bove, Antonio Suppa, Anna Castrioto, Sara Meoni, Pierre Pélissier, Emmanuelle Schmitt, Amélie Bichon, Eugénie Lhommée, Andrea Kistner, Stephan Chabardès, Eric Seigneuret, Valerie Fraix, Elena Moro

**Affiliations:** 1 Division of Neurology, Grenoble Alpes University, CHU of Grenoble, Grenoble Institute of Neurosciences, Inserm U1216, Grenoble, France; 2 Department of Human Neurosciences, Sapienza University of Rome, Rome, Italy; 3 Neurology Unit, Neuromotor and Rehabilitation Department, Azienda USL-IRCCS di Reggio Emilia, Reggio Emilia, Italy; 4 Neurology Unit, Fondazione Policlinico Universitario Agostino Gemelli IRCCS, Rome, Italy; 5 IRCCS Neuromed Institute, Pozzilli, Italy; 6 Division of Neurosurgery, CHU of Grenoble, Grenoble Alpes University, Grenoble, France

**Keywords:** Parkinson's disease, Parkinson's disease

## Abstract

In this retrospective study, we longitudinally analyzed axial impairment and falls in people with Parkinson’s disease (PD) and subthalamic nucleus deep brain stimulation (STN-DBS). Axial scores and falling frequency were examined at baseline, and 1, 10, and 15 years after surgery. Preoperative demographic and clinical data, including PD duration and severity, phenotype, motor and cognitive scales, medications, and vascular changes on neuroimaging were examined as possible risk factors through Kaplan–Meier and Cox regression analyses. Of 302 individuals examined before and at 1 year after surgery, 102 and 57 were available also at 10 and 15 years of follow-up, respectively. Axial scores were similar at baseline and at 1 year but worsened at 10 and 15 years. The prevalence rate of frequent fallers progressively increased from baseline to 15 years. Preoperative axial scores, frontal dysfunction and age at PD onset were risk factors for axial impairment progression after surgery. Axial scores, akinetic/rigid phenotype, age at disease onset and disease duration at surgery predicted frequent falls. Overall, axial signs progressively worsened over the long-term period following STN-DBS, likely related to the progression of PD, especially in a subgroup of subjects with specific risk factors.

## Introduction

Since the early FDA approval of subthalamic nucleus deep brain stimulation (STN-DBS), several randomized clinical trials have documented the efficacy of this treatment for the relief of advanced Parkinson’s disease (PD) symptoms^1–5^. The positive effects of STN-DBS in PD are maintained over time despite the disease progression, thus confirming a favorable risk-benefit ratio also in the long-term period^6–9^. However, the sustained clinical benefits from STN-DBS in PD are undermined by the occurrence of disabling symptoms that are resistant to medical and surgical treatments, including axial impairment and falls^10^. By leading to physical restrictions and injuries, axial impairment (i.e., gait, posture and balance disorders) and falls give rise to a high burden for patients and caregivers and are among the most challenging clinical issues in advanced PD^11^. Indeed, the severity of the axial disability is linked to individual risk of death after DBS surgery^12^, whereas falls are associated with reduced survival and high economic repercussions in PD^11^.

Previous studies have reported inconsistent results concerning the progression of axial signs and the occurrence of falls in the short-term after STN-DBS^13–23^. Moreover, when considering the long-term period, while a few authors agreed on the progressive decline of axial functions up to 10 years after surgery^12,24,25^, no studies have examined the impact of STN-DBS on the occurrence of falls in PD. Hence, a definite consensus concerning the evolution of axial impairment and falls in people with PD after STN-DBS surgery is still absent. A further relevant issue concerns the current lack of preoperative factors predicting axial impairment progression and fall occurrence in the very long-term period after STN-DBS in PD. Indeed, although some authors suggested the preoperative severity of axial impairment and axial sensitivity to L-Dopa as predictors of post-surgical axial disability^12,16,26,27^, these findings have not been consistently replicated in large cohorts of patients. Also, the only study investigating predictors of falls after STN-DBS surgery in PD failed to find significant risk factors^21^. Small sample sizes, short follow-up periods and non-focused experimental designs may have precluded the achievement of firm conclusions on the evolution and predictors of axial impairment and falls after STN-DBS surgery in PD. Accordingly, prolonged longitudinal observations of large samples of individuals with PD after STN-DBS surgery would be necessary to address these issues. The knowledge of the long-term evolution and preoperative risk factors of axial impairment and falls after STN-DBS surgery would help select patients for surgery. Also, it could implement preventive therapeutic strategies to improve axial disability and avoid injuries in PD.

In this retrospective study, we analyzed a large dataset of people with PD and STN-DBS, followed longitudinally up to 15 years after surgery, with the main objectives of evaluating axial signs, including gait, posture and balance disorders, and falls progression and associated risk factors. To fill the gap in the literature, we focused on the very long-term evolution of axial signs and falls in people with PD by evaluating patients 10 and 15 years after STN-DBS surgery under the chronic treatment conditions (i.e., ON stimulation/ON medication). Moreover, in addition to the long-term evaluation, our time-dependent analysis also included a short-term time point (i.e., 1-year follow-up) to examine early changes in axial function and fall occurrence after surgery, supporting the identification of risk factors for these disorders.

## Results

A total of 417 people with PD and bilateral STN-DBS operated between 1993 and 2010 was retrieved from the Movement Disorders Centre database of the Grenoble University Hospital, France. From the analyses, 115 subjects were excluded because of incomplete medical records, surgical complications responsible for persistent neurological sequelae, other brain surgical procedures, or electrode misplacement. Accordingly, based on exclusion criteria and data availability in medical records, 302 people were included with 1-year follow-up (mean ± SD follow-up = 1.03 ± 0.21 years, median = 1.00 year, range = 0.41–3.09 years). There were 102 patients available at 10 years (mean ± SD follow-up = 10.38 ± 1.01 years, median = 10.18 years, range = 7.13–13.06 years) and 57 patients at 15 years (mean ± SD follow-up = 15.32 ± 0.93 years, median = 15.29 years, range = 13.16–17.90 years).

Table 1 shows the main demographic and clinical characteristics of patients at baseline and at follow-up visits after STN-DBS surgery. Detailed preoperative demographic and clinical data of subjects at the different time points after STN-DBS are summarized in Supplementary Table 1.

**Table 1:** Demographic and clinical features of people with Parkinson’s disease and subthalamic deep brain stimulation at baseline and follow-ups.

Variable	Values *n* (%); mean [±SD]; Median {range}			
	Baseline^a^	1-year follow-up^a^	10-year follow-up	15-year follow-up
Patients (*N*) and Sex	302 (183 Male, 60.6%; 119 Female, 39.4%)	302 (183 Male, 60.6%; 119 Female, 39.4%)	102 (63 Male, 61.8%; 39 Female, 38.2%)	57 (36 Male, 63.2%; 21 Female 36.8%)
Age	55.61 [±8.42]; 56 {29.00–74.00}	56.65 [±8.42]; 57 {30.00–74.96}	62.84 [±8.39]; 63 {39.38–80.75}	65.03 [±8.26]; 66 {45.90–83.48}
Disease duration since diagnosis (y)	11.75 [±4.27]; 12.00 {2.00–27.00}	12.75 [±4.33]; 12.94 {2.94–28.09}	22.90 [±4.37]; 22.61 {14.39–34.78}	26.50 [±3.79]; 26.35 {20.28–37.42}
Clinical phenotype	109 AR (36.1%); 40 T (13.2%); 151 Mixed (50.0%)	109 AR (36.1%); 40 T (13.2%); 151 Mixed (50.0%)	37 AR (36.3%); 10 T (9.8%); 55 Mixed (53.9%)	18 AR (31.6%); 6 T (10.5%); 33 Mixed (57.9%)
Hoehn & Yahr	OFF: 3.35 [±0.99]; 3.00 {1.50–5.00} ON: 1.86 [±0.76]; 2.00 {0.00–3.00}	ON stim/ON med: 1.97 [±0.71]; 2.00 {1.00–4.00}	ON stim/ON med: 2.96 [±0.91]; 3.00 {2.00–5.00}	ON stim/ON med: 2.99 [±0.90]; 3.00 {2.00–5.00}
UPDRS-III	OFF: 45.26 [±15.41]; 43.00 {13.00–91.50} ON: 13.76 [±7.98]; 12.00 {1.00–46.00}	ON stim/OFF med: 20.69 [±11.94]; 19 {4.00–63.50} ON stim/ON med: 13.22 [±9.35]; 11 {2.00–53.00}	ON stim/ON med: 29.92 [±15.26]; 27.00 {6.00–79.00}	ON stim/ON med: 35.89 [±17.17]; 32.50 {13.50–90.00}
Axial score	OFF: 6.82 [±3.94]; 6.00 {0.00–16.00} ON: 1.94 [±1.80]; 1.50 {0.00–10.00}	ON stim/ON med: 1.97 [±2.14]; 1.00 {0.00–12.50}	ON stim/ON med: 6.11 [±4.24]; 5.00 {0.00–16.00}	ON stim/ON med: 7.39 [±4.73]; 7.00 {0.00–16.00}
Frequent fallers (*N*)	16 (out of 302; 5.3%)	28 (out of 292; 9.6%)	35 (out of 97; 36.1%)	19 (out of 51; 37.3%)
Frequency of STN-DBS (*N* of subjects with LFS)	NA	130 Hz {60–185 Hz} (4 LFS, 1.32%)	130 Hz {60–185 Hz} (4 LFS, 3.92%)	130 Hz {60–185 Hz} (4 LFS, 7.02%)
LEDDs	1347.67 [±505.86]; 1340 {265.00–3200.00}	495.72 [±392.91]; 400 {0–1796.00}	660.04 [±377.51]; 529 {150.00–1827.00}	662.22 [±305.43]; 645 {200.00–1462}

*AR* akinetic-rigid, *L* left, *LEDDs* L-Dopa equivalent daily doses, *LFS* low frequency stimulation (<100 Hz), *NA* not applicable, *R* right, *STN-DBS* deep brain stimulation of the subthalamic nucleus, *T* tremorigen, *UPDRS* unified Parkinson’s disease rating scale.

^a^All subjects at baseline were also included at the 1-year follow-up.

### Axial impairment and falls after STN-DBS

**Fig. 1:**
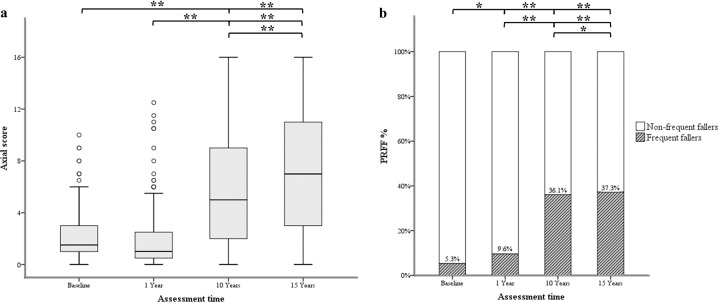
Evolution of axial scores and prevalence rate of frequent-fallers in people with Parkinson’s disease and subthalamic deep brain stimulation. Longitudinal values of axial scores are displayed through box plots (**a**). The central values within boxes correspond to the median (50th percentile, or Q2), whereas the range between the lower (25th percentile, or Q1) and upper (75th percentile, or Q3) bounds of the boxes reflects the interquartile range (IQR). Whiskers include scores outside IQR, whereas ends reflect maximum (Q3 + 1.5 × IQR) and minimum values (Q1 × IQR). Spots are outliers above the maximum values of whiskers. Longitudinal prevalence rate of frequent-fallers (PRFF%) is expressed as a percentage through histograms (**b**). Asterisks indicate the main differences among groups (**p < 0.001, *p < 0.05).

The Friedman test showed significant differences in axial scores at baseline, 1, 10, and 15 years after surgery [*X*^2^(3) = 107.728, *p* < 0.001]. The Wilcoxon signed-rank test demonstrated similar axial scores at baseline and at 1-year follow-up (*Z* = −0.323, *p* = 0.746), but progressively higher axial scores at 10- and 15-year follow-ups (baseline vs. 10-year follow-up: *Z* = −7.712, *p* < 0.001; baseline vs. 15-year follow-up: *Z* = −6.345, *p* < 0.001; 1-year vs. 10-year follow-up: *Z* = −8.195, *p* < 0.001; 1-year vs. 15-year follow-up: *Z* = −6.455, *p* < 0.001; 10-year vs. 15-year follow-up: *Z* = −3.990, *p* < 0.001) (Table 1 and Fig. 1a).

The *χ*^2^ test demonstrated a progressive increase in the number of people with PD presenting frequent falls from baseline through the 15-year follow-up (baseline vs. 1-year follow-up: *χ*^2^(1) = 3.98, *p* = 0.046; baseline vs. 10-year follow-up: *χ*^2^(1) = 62.41, *p* < 0.001; baseline vs. 15-year follow-up: *χ*^2^(1) = 49.89, *p* < 0.001; 1-year vs. 10-year follow-up: *χ*^2^(1) = 37.655, *p* < 0.001; 1-year vs. 15-year follow-up: *χ*^2^(1) = 84.99, *p* < 0.001; 10-year vs. 15-year follow-up: *χ*^2^(1) = 9.59, *p* < 0.01) (Table 1 and Fig. 1b).

### Risk factors for axial impairment and falls

Preoperative independent variables selected through the univariate analysis and entering the stepwise selection process are reported in Table 2.

**Table 2:** Univariate analysis for variable selection (*p* < 0.20) and multivariable Cox regression analysis for risk factors.

	Univariate analysis			Multivariable Cox regression		
	HR	95% CI (lower–upper)	*p* value	HR	95% CI (lower–upper)	*p* value
Axial impairment						
Age at disease onset	1.058	1.025–1.093	<0.001	1.095	1.044–1.148	**<0.001**
Disease duration at surgery	1.090	1.035–1.148	0.001	0.978	0.889–1.076	0.649
Clinical phenotype (AR vs. tremorigen and mixed)	0.521	0.325–0.834	0.007	0.716	0.358–1.434	0.346
WMH on brain MRI	2.595	1.340–5.024	0.005	1.715	0.820–3.585	0.152
Baseline UPDRS part III OFF medication	1.017	1.002–1.032	0.030	0.992	0.959–1.026	0.638
Baseline UPDRS part III ON medication	1.053	1.028–1.078	<0.001	1.002	0.949–1.059	0.931
Baseline H&Y OFF medication	1.587	1.223–2.061	0.001	1.127	0.626–2.030	0.690
Baseline H&Y ON medication	1.393	1.007–1.926	0.045	0.660	0.395–1.103	0.113
Baseline axial score OFF medication	1.179	1.104–1.258	<0.001	1.104	0.980–1.243	0.103
Baseline axial score ON medication	1.544	1.373–1.736	<0.001	1.468	1.217–1.770	**<0.001**
Baseline MDRS	0.955	0.912–0.999	0.045	1.064	0.985–1.150	0.114
Baseline frontal score	0.935	0.901–0.969	<0.001	0.952	0.909–0.998	**0.042**
Baseline L-Dopa responsiveness of axial signs	0.987	0.979–0.996	0.005	1.007	0.982–1.033	0.582
Falls						
Age at disease onset	1.037	1.008–1.067	0.013	1.051	1.011–1.092	**0.011**
Disease duration at surgery	1.097	1.046–1.152	<0.001	1.117	1.117–1.045	**0.001**
Clinical phenotype (AR vs. tremorigen and mixed)	1.839	1.184–2.856	0.007	1.869	1.114–3.135	**0.018**
Baseline UPDRS part III ON medication	1.026	1.000–1.052	0.047	0.986	0.949–1.025	0.489
Baseline H&Y OFF medication	1.368	1.085–1.725	0.008	0.932	0.581–1.494	0.769
Baseline H&Y ON medication	1.497	1.086–2.063	0.014	1.085	0.710–1.659	0.706
Baseline axial score OFF medication	1.126	1.062–1.194	<0.001	1.045	0.956–1.142	0.336
Baseline axial score ON medication	1.382	1.242–1.537	<0.001	1.291	1.133–1.471	**<0.001**
Baseline L-Dopa responsiveness of axial signs	0.989	0.980–0.997	0.009	0.996	0.975–1.018	0.733
Baseline frontal score	0.974	0.943–1.007	0.117	1.022	0.985–1.060	0.243

Bold font indicates statistical significance of the variable at the Cox regression analysis.

*AR* akinetic/rigid, *CI* confidence interval, *H&Y* Hoehn and Yahr scale, *HR* hazard ratio, *MDRS* Mattis dementia rating scale, *UPDRS* unified Parkinson’s disease rating scale, *WMH* white matter hyperintensities.

**Fig. 2:**
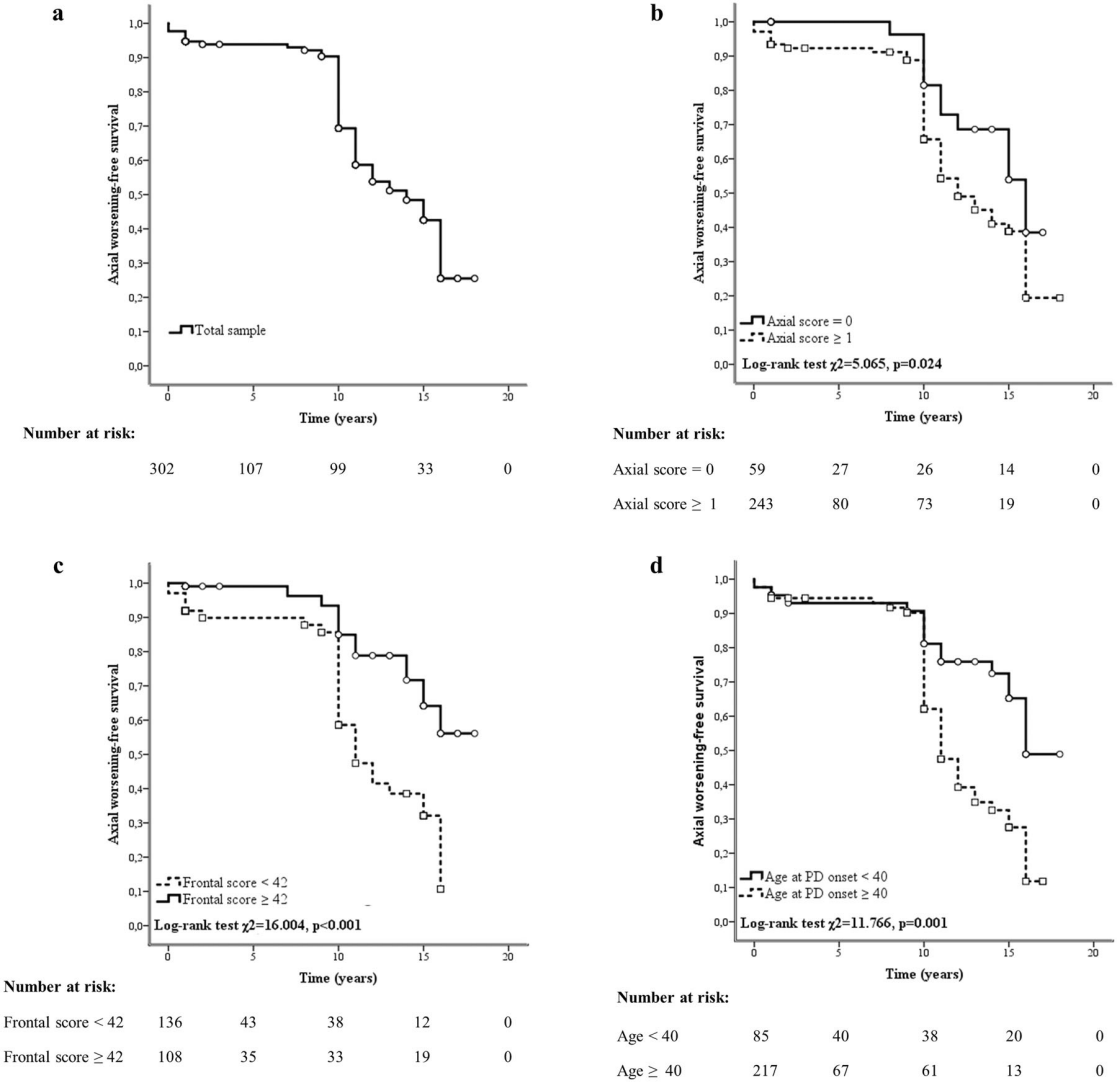
Risk factors for axial impairment progression in people with Parkinson’s disease and subthalamic deep brain stimulation. Kaplan–Meier curves show axial impairment progression in the total sample (**a**) and subgroups of people with Parkinson’s disease (PD) and subthalamic deep brain stimulation based on preoperative axial scores (**b**), frontal scores (**c**), and age at disease onset (**d**). The “axial worsening-free survival” reflects axial impairment progression intended as the achievement of an axial score ≥7. Squares and circles in the Kaplan–Meier curves indicate censored data.

The multivariable Cox regression analysis showed that higher axial scores in the ON medication condition (adjusted hazard ratio (HR) 1.468), lower frontal scores (HR 0.952), and higher age at PD onset (HR 1.095) acted as risk factors for axial impairment progression after STN-DBS (Table 2). Figure 2 illustrates Kaplan–Meier curves displaying the axial impairment progression in the total sample and subgroups of individuals based on the identified risk factors, with the related log-rank tests.

**Fig. 3:**
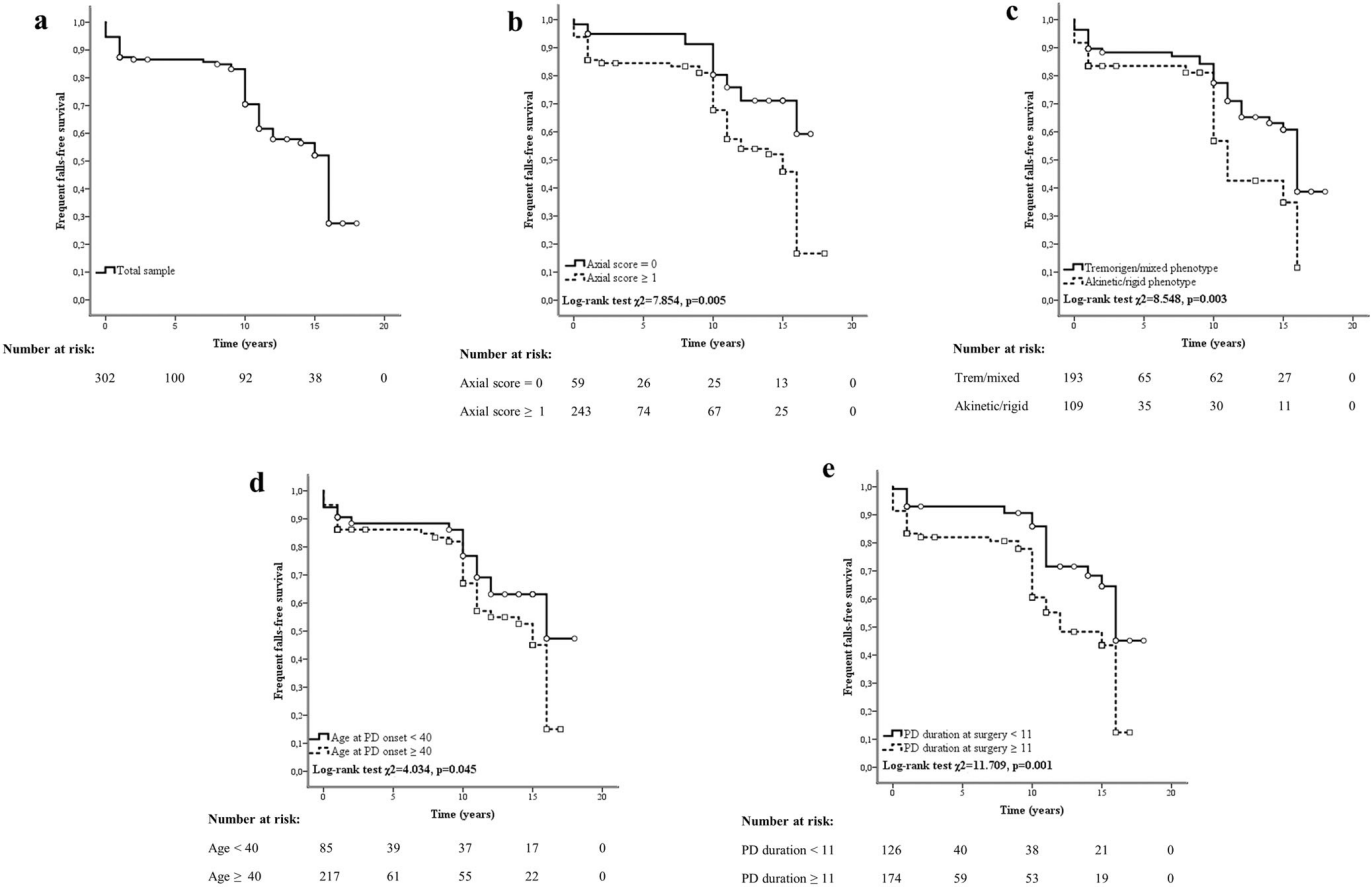
Risk factors for frequent falls in people with Parkinson’s disease and subthalamic deep brain stimulation. Kaplan–Meier curves show the occurrence of frequent falls in the total sample (**a**) and subgroups of people with Parkinson’s disease (PD) and subthalamic deep brain stimulation based on preoperative axial scores (**b**), clinical phenotype (**c**), age at disease onset (**d**), and disease duration at surgery (**e**). The “frequent falls-free survival” reflects the occurrence of frequent falls based on item 13 of the Unified Parkinson’s Disease Rating Scale part II ≥ 2. Squares and circles in the Kaplan–Meier curves indicate censored data.

Concerning falls, higher axial scores in the ON medication condition (HR 1.291), akinetic/rigid phenotype (HR 1.869), higher age at PD onset (HR 1.051), and longer disease duration at surgery (HR 1.117) acted as risk factors for frequent falls after STN-DBS (Table 2). In Fig. 3, Kaplan–Meier curves display the rate of frequent fallers in the total sample and subgroups of individuals based on the identified risk factors, with the related log-rank tests.

### Clinical correlations

The Spearman’s rank correlation test showed that axial scores and the “frequent faller” condition (i.e., increased falling incidence based on item 13 of the UPDRS part II ≥ 2) correlated at baseline (rho = 0.25, *p* < 0.001), at 1-year (rho = 0.23, *p* < 0.001), at 10-year (rho = 0.43, *p* < 0.001), and at 15-year follow-ups (rho = 0.34, *p* = 0.015). Frequency parameter of STN-DBS was not associated with axial scores at any time points as well as with the “frequent faller” condition at baseline, at 1- and at 10-year follow-ups (*p* > 0.05). Conversely, frequency parameter of stimulation was inversely correlated with the “frequent faller” condition at 15 years (left STN-DBS: rho = −0.32, *p* = 0.026; right STN-DBS: rho = −0.28, *p* = 0.05).

## Discussion

By considering a large dataset of people with PD and STN-DBS followed longitudinally up to 15 years, we found stable axial scores at 1 year after surgery, but a progressive decline in the long-term period, at 10 and 15 years, in the chronic ON stimulation/ON medication condition. We also found a progressive increase in the prevalence rate of frequent fallers from the preoperative evaluation to the 15-year postoperative follow-up. Finally, we identified several risk factors for axial impairment progression and frequent falls after surgery, such as the preoperative axial disability, frontal dysfunction severity and older age at PD onset.

This study involved the largest sample of patients so far reported concerning axial impairment and falls in the long-term follow-up after STN-DBS in PD. Moreover, the longest longitudinal observation of axial impairment progression and fall occurrence, up to 15 years after surgery, has been here reported. The large sample size and prolonged follow-up have allowed to reach higher statistical power in the investigation of axial impairment and fall occurrence compared to the available literature, as well as to prevent potential confounders in the survival analysis by adjusting for different periods of observation and several clinical variables in the Cox proportional hazards models. The present study also examined the direct relationship between axial impairment and falls in people with PD undergoing STN-DBS surgery.

When assessing axial impairment in the short-term period, axial scores at 1 year after STN-DBS were comparable with preoperative evaluation in the ON medication condition. This finding is fully in line with previous studies showing unchanged axial functions in the short-term follow-up after STN-DBS in PD^15,18,25,28,29^, thus excluding a detrimental impact of STN-DBS on axial impairment up to 1 year after surgery. Our results are in contrast with those reporting short-term improvement or worsening of axial functions after STN-DBS^13,16,17,19,20^. These inconsistencies likely reflect the different clinical approaches adopted to assess axial impairment through heterogeneous clinical scales and scores, sometimes simultaneously involving axial signs with different underlying pathophysiological mechanisms, such as speech and postural disorders^17,20,30,31^. Conversely, we have measured the axial score as the sum of specific items from the Unified Parkinson’s Disease Rating Scale (UPDRS) part III only directly referred to static and dynamic postural control, thus excluding other axial signs, like voice and facial expression disorders, possibly representing confounding factors in the interpretation of data.

Concerning the very long-term period, the worsening of axial functions in our patients confirms findings from previous studies with shorter follow-ups (i.e., from 4 to 10 years)^13,16,18,25,26,32^. These data confirm the progressive decline of axial functions in PD in the long-term period, likely resulting from the natural history of the disease, as reported in non-operated patients^33,34^. Although not conclusive, the lack of correlations between the frequency parameter of stimulation and the axial scores is in line with this hypothesis. Overall, the evidence of progressive axial impairment over the long-term period despite STN-DBS supports the current consensus criteria for DBS in PD that recommend to not consider for surgery individuals suffering from L-Dopa unresponsive axial symptoms^35^. Also, these findings further emphasize the need for predictive factors of axial impairment progression to properly select patients undergoing STN-DBS and promptly perform preventive strategies.

The prevalence rate of frequent fallers in our cohort increased progressively over time after surgery. Our findings are in line with those from a recent study that found increased fall frequency 1 year after STN-DBS in a large sample of people with PD^21^. Conversely, the lack of significant changes in the short-term rate of falls after STN-DBS reported in some previous studies could be attributed to the limited number of patients enrolled (i.e., <40 subjects)^15,22,23^. The present study also investigated fall occurrence at 10 and 15 years after STN-DBS, showing a progressive increase in the number of subjects suffering from frequent falls. Several possible mechanisms may contribute to this progressive worsening of fall frequency after STN-DBS. Given the unchanged axial scores at baseline and at 1-year follow-up, it is unlikely that postural and gait disorders are responsible for the increased prevalence rate of frequent fallers in the short term. Accordingly, people with PD might fall more frequently in the short-term period after STN-DBS owing to improved motor performances and increased activity levels providing new opportunities for falling^2^. Conversely, the progressive decline of axial functions would play a relevant role in fall severity in the long-term period. Indeed, in line with this hypothesis, the correlation between axial scores and fall occurrence was stronger at 10 and 15 years than at baseline and at 1-year follow-up. Also, the “frequent faller” condition inversely correlated with the frequency parameter of STN-DBS at 15 years, possibly reflecting clinical efforts to improve axial signs through lower stimulation frequencies^36,37^. Lastly, the percentage of subjects presenting frequent falls at 15 years (i.e., 37.3%) was slightly but significantly higher than at 10 years after surgery (i.e., 36.1%), in accordance with the “inverted u-shaped curve” model of falls in PD^38^. This model predicts that the number of falls in people with PD progressively increases as motor conditions deteriorate, up to the occurrence of severe physical restrictions confining subjects to wheelchairs or beds and, thus, limiting the falling frequency^38^.

People with PD presenting at baseline with a more severe axial disability, frontal cognitive dysfunction, and older age at the disease onset showed a mildly increased risk for axial impairment progression after STN-DBS surgery. Accordingly, we here confirm the relevant role of preoperative axial disability in predicting the progression of axial signs after STN-DBS^16,26^. Although axial responsiveness to L-Dopa did not reach a statistical significance in our predictive model, the association of axial impairment progression with axial scores in ON medication, but not in OFF medication, supports the hypothesis that the lower improvement of axial signs under dopaminergic stimulation involves a higher risk for axial disability progression, in agreement with previous observations^12,29^. Accordingly, in PD, the increasing impairment of static and dynamic postural functions likely reflects the progressive involvement of extranigral pathways which remain unaffected by L-Dopa and STN-DBS^39^.

The present study suggests that higher frontal abilities may act as a protective factor for axial impairment progression in people with PD and STN-DBS. This finding confirms the well-known impact of cognitive functions, primarily concerning frontal executive abilities, on balance and gait^40^. Indeed, people with PD typically show worsened balance and gait during dual-task paradigms as a result of reduced cognitive reserve and increased attentional demand for motor performances^40^. Further supporting the strict relationship between axial impairment and cognitive dysfunction in PD^40^, our results highlight the need for strict selection criteria when considering STN-DBS in people with PD by including those with preserved executive abilities to maximize the opportunity for long-term benefits after surgery.

Lastly, our analysis suggests that higher age at PD onset would contribute as an additional risk factor to axial impairment progression in people with PD and STN-DBS. This result is in line with a previous study showing early deterioration of axial signs in individuals with advanced age at surgery^27^. Moreover, it expands previous observations of a better general motor outcome in younger patients undergoing STN-DBS by confirming similar conclusions concerning axial functions^41^. In addition to disease-specific issues, several age-related factors (e.g., cardiovascular co-morbidities and polypharmacy) could contribute to deteriorating cognitive and motor performances in PD, resulting in increased severity of axial signs in older individuals with STN-DBS.

In our cohort of people with PD and STN-DBS, preoperative severity of axial disability (i.e., axial scores in the ON medication condition), akinetic/rigid phenotype, age at disease onset and PD duration at surgery were associated with a mildly increased risk for frequent falls after surgery. Previous studies similarly reported abnormal axial functions and akinetic/rigid phenotype as predictors of falls in people with PD under medical treatment^42,43^. Indeed, axial impairment and akinetic/rigid phenotype are strictly linked in PD and are both associated with increased cognitive decline and malignant course of the disease compared to patients with preserved axial abilities and tremor-dominant appearance^44^. This study replicates the same conclusions in patients undergoing surgery, thus suggesting that these pathophysiological mechanisms underlying falls are unrelated to STN-DBS in PD.

Besides the prediction of axial impairment, higher age at PD onset was also associated with frequent falls after STN-DBS. This finding is in line with previous observations that older people with PD are usually those with earlier onset of falls and faster disease progression compared to younger subjects^44^. Indeed, in a community-living healthy population, more than one-third of subjects older than 65 falls each year, further pointing to the harmful synergy between disease-related issues and ageing in PD. Hence, although no specific age cut-off has been defined for STN-DBS, particular care should be taken when considering surgery for older individuals and prudently analyze the benefits-to-risks ratio^35^.

A final comment concerns our observation of a longer PD duration at surgery as a possible risk factor for frequent falls after STN-DBS. Although longer disease duration has been previously found as a predictor of a positive outcome for STN-DBS^45^, it is also associated with more severe clinical symptoms likely explaining an increased occurrence of falls. Therefore, STN-DBS may have greater beneficial effects for patients with less advanced PD by limiting the incidence of falls and allowing sustained maintenance of individual autonomy^5^.

When considering the present study, some limitations should be taken into account. Although using a standardized item of the UPDRS part II (i.e., item 13), the evaluation of falls can be affected by “recall bias” that would underestimate the frequency of this issue in people with PD and STN-DBS. Also, medical records did not allow to classify falls according to severity and associated injuries, possibly limiting the interpretation of findings. Similarly, incomplete medical records concerning white matter hyperintensities (WMH) on brain magnetic resonance imaging (MRI) in a high percentage of people with PD (i.e., up to 34.3% of subjects at the 10-year follow-up) may have overlooked the prognostic role of this variable on the evolution of axial impairment and falls after STN-DBS surgery. Moreover, like in previous longitudinal studies involving patients undergone surgery^12,24^, the lack of a control group precluded the direct comparison of the evolution of axial signs and fall occurrence in subjects with and without STN-DBS. Accordingly, some of the conclusions of this study were based on the indirect comparison with the natural history of the disease previously reported in cohorts of non-operated subjects^33,34^. Owing to the very long-term longitudinal observation of people with PD and STN-DBS, this study was unavoidably burdened by a high percentage of subjects lost to follow-up, possibly underestimating the incidence of axial impairment and falls after STN-DBS surgery. Lastly, the clinical evaluation of patients at 10- and 15-year follow-ups was performed under the chronic treatment conditions (i.e., ON stimulation/ON medication), thus precluding the assessment of the isolated effects of the STN-DBS on acute axial impairment and acute fall occurrence. Nevertheless, according to the prominent postoperative LEDD reduction compared to the preoperative period as well as dopamine receptor desensitization following continuous STN stimulation^46,47^, it is likely that differences between the ON and OFF medication conditions under DBS stimulation would be minimal. This dopamine desensitization is, indeed, particularly evident in people with long-term STN-DBS, in whom the sudden lack of stimulation can be fatal and clinical improvement not achieved even by very high doses of L-Dopa (the so called life-threatening DBS withdrawal syndrome)^48^. Finally, it should be considered that axial dysfunction becomes progressively refractory to L-Dopa^49^, further suggesting that also STN-DBS would lose its beneficial effects on these signs over time. Overall, the assessment of axial impairment and falls in the chronic treatment conditions would reflect real-life patients’ status, and thus provide ecologic information on these signs in our cohort of people with PD.

In conclusion, although axial function in PD remains stable in the short term after STN-DBS surgery, it progressively worsens in the long-term period, likely as a result of the natural history of the disease. Conversely, STN-DBS may be indirectly involved in the increased occurrence of falls since the first year after surgery by improving motor performance and leading to increased activity levels. Specific motor, cognitive and demographic features at baseline, including axial disability, frontal dysfunction and age at PD onset, can act as possible risk factors for axial impairment progression after STN-DBS. Similarly, axial disability, akinetic/rigid phenotype, age at disease onset and disease duration at surgery would contribute to the occurrence of frequent falls in people with PD and STN-DBS. Therefore, in the selection procedures for STN-DBS, it would be relevant to consider these factors to provide patients and their caregivers with reasonable prognostic information and maximize the long-term outcomes of candidates for surgery. Our study focused on the preoperative features predicting axial impairment progression and falls in PD people with STN-DBS. Future studies will clarify the potential impact of postoperative variables on these disorders in PD, including factors related to active lead contact and stimulation parameters.

## Methods

This study was conducted in accordance with the “Strengthening the Reporting of Observational studies in Epidemiology” guidelines (Supplementary Data 1)^50^.

### Subjects

Consecutive subjects with PD who underwent bilateral STN-DBS at the Grenoble Alpes University Hospital, France, between 1993 and 2010 were retrospectively included in this cohort study. DBS surgery was performed according to accepted indications and procedures, as well as standard stereotactic techniques^51^. All subjects received postoperative neuroimaging to verify the post-implantation placement of leads. Moreover, an expert in movement disorders periodically optimized DBS programming parameters, including intensity, frequency, pulse width and contact configuration, according to patients’ motor symptoms. The selection criteria for surgery included the clinical diagnosis of idiopathic PD according to the UK Brain Bank, the occurrence of motor complications despite optimized antiparkinsonian medications, absence of dementia, major ongoing psychiatric illness and surgical contraindications. People with previous neurosurgical brain interventions other than STN-DBS, surgical complications, implantation of more than two electrodes or involvement of nuclei other than the STN, and electrode misplacement (i.e., suboptimal electrode’s location requiring lead revision) were excluded from this study. All subjects provided written informed consent for data collection and use for research purposes. The institutional research centre authority of the Grenoble Alpes University Hospital approved the study protocol.

### Clinical assessments

Data were collected from clinical evaluations at baseline and 1, 10, and 15 years after STN-DBS surgery. Demographic and clinical data, including age, sex, age at disease onset, disease duration at surgery, phenotype, and presence of WMH of vascular origin on brain MRI based on a semi-quantitative visual assessment through the Fazekas’ scale^52^, were collected at baseline. The preoperative assessment was performed in the OFF and ON medication conditions and included the following clinical scales: UPDRS^53^, Hoehn and Yahr scale (H&Y)^54^, Mattis Dementia Rating Scale (MDRS)^55^, frontal score (maximal score of 50 points including the following: up to 20 points from the number of criteria established in the Wisconsin card sorting test × 3, +2 points if the number of responses needed is <43; up to 10 points from the verbal fluency test scores/3; up to 20 points from the graphic and motor series)^56^ and Beck Depression Inventory-II^57^. The postoperative assessment included the UPDRS (up to 2011) or Movement Disorder Society-sponsored revision of the UPDRS (from 2012)^58^ in the ON stimulation/OFF and ON medication conditions at 1 year, and in the chronic ON stimulation/ON medication condition at 10- and 15-year follow-ups. Axial impairment was assessed through the axial score, ranging from 0 to 16, intended as the sum of the items 27 or 3.9 (i.e., arising from the chair), 28 or 3.13 (i.e., posture), 29 or 3.10 (i.e., gait) and 30 or 3.12 (i.e., postural stability) of the UPDRS or MDS-UPDRS, respectively^16,18^. Concerning the assessment of falls, item 13 (“falls in the absence of FOG”) of the UPDRS part II, with scores ranging from 0 to 4 based on the falling frequency (i.e., 0–1 no or rare falls; 2–4 frequent or habitual falls) was used. Finally, the L-Dopa equivalent daily doses (LEDDs) were calculated in people with PD before and after STN-DBS according to standardized procedures^59^.

### Statistical analysis

The primary outcome consisted of changes in the axial score and prevalence rate of frequent fallers at baseline, 1-, 10- and 15-year follow-ups after STN-DBS surgery. The Friedman and Wilcoxon signed-rank tests were used for comparing axial scores at baseline and follow-ups in the best treatment conditions (i.e., ON medication at baseline; ON stimulation/ON medication at follow-ups). The *χ*^2^ test was used to compare the prevalence rate of frequent fallers by considering the number of people reporting frequent falls based on item 13 of the UPDRS part II ≥ 2 (i.e., “frequent faller” condition) when in ON medication at baseline and ON stimulation/ON medication at follow-ups.

The secondary outcome was the evaluation of risk factors for axial impairment progression and frequent falls after surgery. Multivariable Cox regression analysis was used to simultaneously assess the effect of preoperative independent variables. Concerning axial impairment progression, for statistical purposes, an event was defined as the achievement of the median value of the axial score in the most impaired patients (i.e., subjects at 15 years after surgery), reflecting a clinically meaningful worsening of axial signs (i.e., axial score ≥7). Univariate analysis was first used to examine the association of preoperative independent variables with the outcome variables and determine factors providing the best fit for prediction models. Preoperative independent variables showing significance with *p* < 0.20 in the univariate analysis entered the stepwise selection process for the final building of multivariable Cox regression models, in line with previously reported procedures^60–62^. To rule out collinearity and lack of independence among variables, pairwise correlations were checked among key covariates and those presenting strong reciprocal associations were excluded from the analysis. A threshold of 0.7 was considered to exclude strong associations among the independent variables, as previously suggested^63,64^. Kaplan–Meier product-limit method was utilized to display the “axial worsening-free survival” (i.e., axial impairment progression intended as the achievement of an axial score ≥7) and the “frequent falls-free survival” (i.e., occurrence of frequent falls based on item 13 of the UPDRS part II ≥ 2) in the total sample and subgroups of patients based on identified risk factors. To distinguish subgroups of patients, we divided subjects based on the presence of categorical risk factors (e.g., subjects with or without WMH). Alternatively, in the case of risk factors consisting of continuous variables (e.g., axial scores), the median baseline scores in the most impaired subjects (i.e., patients at 15 years after surgery) were considered cut-off values for discriminating subgroups (e.g., axial scores ≥1). The log-rank test was also performed for each significant variable entered the final Cox models. Concerning candidate risk factors, the following independent variables were assessed: age at disease onset, disease duration at surgery, phenotype (i.e., akinetic/rigid, tremorigen, mixed), presence of WMH on brain MRI, UPDRS part III (OFF and ON conditions), H&Y (OFF and ON conditions), axial score (OFF and ON conditions), L-Dopa responsiveness of axial signs, MDRS, frontal score, and LEDDs. Survival analysis statistics were calculated from baseline to outcome achievement or censored data at the last available follow-up. HR with a 95% confidence interval was also measured. The pairwise deletion method was adopted to handle missing data.

Finally, Spearman’s rank correlation test was utilized to investigate the relationship between axial scores and the “frequent faller” condition at baseline and follow-ups. Spearman’s rank correlation test was also used to examine the possible association of axial scores and the “frequent faller” condition with the frequency parameter of STN-DBS. For all statistical tests, the significant level was set at 0.05 (two-tailed).

Statistical analysis was performed with the SPSS package (IBM-SPSS Inc., USA).

## Supplementary information

Supplementary Information

## Data Availability

The dataset analyzed during the current study is available from the corresponding author upon reasonable request (e.g., reproducibility of research). Sharing restrictions will be applied to sensitive data for privacy-preserving purposes.
