# Supplementary material for: Axial impairment and falls in Parkinson’s disease: 15 years of subthalamic deep brain stimulation
Source: NPJ Parkinsons Dis. 2022 Sep 24;8:121. doi: 10.1038/s41531-022-00383-y (PMC9509398; doi:10.1038/s41531-022-00383-y)
Supplement: Supplementary file 1 — Supplementary Information [file 41531_2022_383_MOESM1_ESM.pdf]

Supplementary Table 1. Preoperative demographic and clinical features of people with Parkinson's disease and subthalamic deep brain stimulation at baseline and follow-ups

Variable	Values n (%); mean [±SD]; Median {range}		
	**Baseline and 1-year follow-up[1]**	**10-year follow-up**	**15-year follow-up**
**Patients (N) and Sex**	302 (183 Male, 60.6%; 119 Female, 39.4%)	102 (63 Male, 61.8%; 39 Female, 38.2%)	57 (36 Male, 63.2%; 21 Female 36.8%)
**Age at disease onset**	43.96 [±8.18]; 44 {19.00-62.00}	40.77 [±7.64]; 41 {24.00-61.00}	39.67 [±7.16]; 40 {24.00-56.00}
**Age at surgery (y)**	55.61 [±8.42]; 56.00 {29.00-74.00}	53.36 [±8.47]; 53.50 {31.00-70.00}	50.79 [±8.33]; 53.00 {31.00-69.00}
**Disease duration at surgery (y)**	11.75 [±4.27]; 12.00 {2.00-27.00}	12.41 [±4.37]; 12.00 {4.00-24.00}	11.17 [±3.68]; 11.00 {5.00-21.00}
**Clinical phenotype**	109 AR (36.1%); 40 T (13.2%); 151 Mixed (50.0%)	37 AR (36.3%); 10 T (9.8%); 55 Mixed (53.9%)	18 AR (31.6%); 6 T (10.5%); 33 Mixed (57.9%)
**WMH on brain MRI**	No 195 (64.6%); Yes 37 (12.3%); Missing data 70 (23.2%)	No 59 (57.8%); Yes 8 (7.8%); Missing data 35 (34.3%)	No 45 (78.9%); Yes 7 (12.3%); Missing data 5 (8.8%)
**Baseline UPDRS-I**	1.94 [±1.57]; 2.00 {0.00-8.00}	1.98 [±1.53]; 2.00 {0.00-7.00}	2.03 [±1.41]; 2.00 {0.00-5.00}
**Baseline UPDRS-II**	OFF: 24.41 [±7.32]; 24.00 {3.00-42.00}<br>ON: 5.87 [±4.54]; 5.00 {0.00-23.50}	OFF: 24.50 [±6.82]; 25.00 {8.00-40.00}<br>ON: 5.52 [±4.35]; 4.50 {0.00-23.50}	OFF: 23.77 [±6.52]; 24.00 {8.00-37.00}<br>ON: 5.02 [±4.26]; 4.00 {0.00-15.00}
**Baseline UPDRS-III**	OFF: 45.26 [±15.41]; 43.00 {13.00-91.50}<br>ON: 13.76 [±7.98]; 12.00 {1.00-46.00}	OFF: 47.08 [±15.91]; 47.25 {15.50-91.50}<br>ON: 13.06 [±8.17]; 11.25 {1.00-45.00}	OFF: 43.42 [±12.83]; 43.50 {15.50-71.00}<br>ON: 10.93 [±6.77]; 9.00 {1.00-30.00}
**Baseline UPDRS-IV**	9.54 [±3.14]; 9.00 {0.00-17.00}	9.76 [±3.25]; 10.00 {3.00-17.00}	10.44 [±3.13]; 11.00 {3.00-16.00}
**Baseline Hoehn & Yahr**	OFF: 3.35 [±0.99]; 3.00 {1.50-5.00}<br>ON: 1.86 [±0.76]; 2.00 {0.00-3.00}	OFF: 3.42 [±0.91]; 4.00 {2.00-5.00}<br>ON: 1.70 [±0.77]; 2.00 {0.00-3.00}	OFF: 3.25 [±0.87]; 3.00 {2.00-5.00}<br>ON: 1.55 [±0.77]; 2.00 {0.00-2.50}
**Baseline axial score**	OFF: 6.82 [±3.94]; 6.00 {0.00-16.00}<br>ON: 1.94 [±1.80]; 1.50 {0.00-10.00}	OFF: 6.65 [±3.44]; 6.00 {0.00-16.00}<br>ON: 1.67 [±1.65]; 1.00 {0.00-8.00}	OFF: 5.53 [±2.64]; 5.00 {0.00-12.00}<br>ON: 1.22 [±1.15]; 1.00 {0.00-5.00}
**Baseline axial response to L-Dopa (%)**	69.50 [±23.79]; 71.00 {0.00-100.00}	74.26 [±23.52]; 80.00 {0.00-100.00}	76.73 [±21.79]; 81.50 {25.00-100.00}
**Baseline MDRS**	137.43 [±5.04]; 139.00 {122.00-144.00}	137.16 [±4.93]; 139.00 {124.00-144.00}	137.41 [±5.08]; 139.00 {125.00-144.00}
**Baseline frontal score**	38.96 [±7.87]; 40.00 {17.60-50.00}	39.99 [±6.82]; 40.05 {26.00-50.00}	40.82 [±6.87]; 42.00 {26.00-50.00}
**Baseline BDI-II**	11.58 [±7.36]; 10.00 {0.00-42.00}	11.67 [±7.31]; 10.00 {0.00-37.00}	12.17 [±7.08]; 11.00 {0.00-37.00}
**Baseline LEDDs**	1347.67 [±505.86]; 1340.00 {265.00-3200.00}	1375.64 [±427.13]; 1364.00 {554.00-2479.00}	1341.24 [±436.92]; 1250.00 {555.00-2347.00}

AR: akinetic-rigid; BDI: Beck depression inventory; LEDDs: L-Dopa equivalent daily doses; MDRS: Mattis dementia rating scale; MRI: magnetic resonance imaging; OFF: not under dopaminergic therapy; ON: under dopaminergic therapy; T: tremorigen; UPDRS: unified Parkinson's disease rating scale; WMH: white matter hyperintensities. [1]All subjects at baseline were also included at the 1-year follow-up.

Supplementary Data 1. STROBE Statement - checklist of items for reports of cohort studies

	Item No	Recommendation	Page No
**Title and abstract**	1	(*a*) Indicate the study's design with a commonly used term in the title or the abstract<br>(*b*) Provide in the abstract an informative and balanced summary of what was done and what was found	3 (a,b)
**Introduction**			
Background/rationale	2	Explain the scientific background and rationale for the investigation being reported	4-5
Objectives	3	State specific objectives, including any prespecified hypotheses	5
**Methods**			
Study design	4	Present key elements of study design early in the paper	5
Setting	5	Describe the setting, locations, and relevant dates, including periods of recruitment, exposure, follow-up, and data collection	14
Participants	6	(*a*) Give the eligibility criteria, and the sources and methods of selection of participants. Describe methods of follow-up<br>(*b*) For matched studies, give matching criteria and number of exposed and unexposed	14-15 (a)
Variables	7	Clearly define all outcomes, exposures, predictors, potential confounders, and effect modifiers. Give diagnostic criteria, if applicable	14-16
Data sources/ measurement	8*	For each variable of interest, give sources of data and details of methods of assessment (measurement). Describe comparability of assessment methods if there is more than one group	14-17
Bias	9	Describe any efforts to address potential sources of bias	14-17
Study size	10	Explain how the study size was arrived at	5, 14
Quantitative variables	11	Explain how quantitative variables were handled in the analyses. If applicable, describe which groupings were chosen and why	14-17
Statistical methods	12	(*a*) Describe all statistical methods, including those used to control for confounding<br>(*b*) Describe any methods used to examine subgroups and interactions<br>(*c*) Explain how missing data were addressed<br>(*d*) If applicable, explain how loss to follow-up was addressed<br>(*e*) Describe any sensitivity analyses	14-17 (a,b,c)
**Results**			
Participants	13*	(a) Report numbers of individuals at each stage of study—eg numbers potentially eligible, examined for eligibility, confirmed eligible, included in the study, completing follow-up, and analysed<br>(b) Give reasons for non-participation at each stage<br>(c) Consider use of a flow diagram	5-6 (a,b)
Descriptive data	14*	(a) Give characteristics of study participants (eg demographic, clinical, social) and information on exposures and potential confounders<br>(b) Indicate number of participants with missing data for each variable of interest<br>(c) Summarise follow-up time (eg, average and total amount)	5-7 (a,b,c)
Outcome data	15*	Report numbers of outcome events or summary measures over time	5-7

Main results	16	(*a*) Give unadjusted estimates and, if applicable, confounder-adjusted estimates and their precision (eg, 95% confidence interval). Make clear which confounders were adjusted for and why they were included<br>(*b*) Report category boundaries when continuous variables were categorized<br>(*c*) If relevant, consider translating estimates of relative risk into absolute risk for a meaningful time period	5-7 (a,b,c)
Other analyses	17	Report other analyses done—eg analyses of subgroups and interactions, and sensitivity analyses	7
**Discussion**			
Key results	18	Summarise key results with reference to study objectives	7-8
Limitations	19	Discuss limitations of the study, taking into account sources of potential bias or imprecision. Discuss both direction and magnitude of any potential bias	12-13
Interpretation	20	Give a cautious overall interpretation of results considering objectives, limitations, multiplicity of analyses, results from similar studies, and other relevant evidence	7-14
Generalisability	21	Discuss the generalisability (external validity) of the study results	7-14
**Other information**			
Funding	22	Give the source of funding and the role of the funders for the present study and, if applicable, for the original study on which the present article is based	18
